# Minimally invasive, robotic-assisted resection of abdominal wall tumors: a case series

**DOI:** 10.3389/fsurg.2026.1779371

**Published:** 2026-05-20

**Authors:** Johannes Maria Alberto Toti, Ramon Pini, Sebastiano Spampatti, Lorenzo Bernardi, Barbara Kopf, Francesco Mongelli, Fabio Garofalo, Fabiano Iaquinandi, Davide La Regina

**Affiliations:** 1 Department of Surgery, Ospedale Regionale di Bellinzona e Valli, EOC, Bellinzona, Switzerland; 2 Department of Surgery, Ospedale Regionale di Lugano, EOC, Lugano, Switzerland; 3 Istituto Oncologico Della Svizzera Italiana (IOSI), Ospedale San Giovanni, Bellinzona, Switzerland; 4 Faculty of Biomedical Sciences, Università Della Svizzera Italiana, Lugano, Switzerland

**Keywords:** abdominal wall reconstruction, abdominal wall tumors, mesh reconstruction, minimally invasive surgery, R0 resection, robotic surgery

## Abstract

**Background:**

The surgical management of abdominal wall tumors poses a dual challenge: achieving oncologically sound resection while preserving abdominal wall integrity. Although traditionally approached via open surgery, robotic-assisted techniques offer promising advantages in selected patients. The aim of this study was to evaluate the feasibility, safety, and short-term outcomes of robotic-assisted resection of abdominal wall tumors confined to the musculofascial layer.

**Methods:**

We retrospectively analyzed five patients who underwent robotic-assisted resection of abdominal wall tumors at a tertiary center between January 2020 and April 2025. All resections were performed using the da Vinci Xi robotic system. Demographic, clinical, surgical, and postoperative data were collected and analyzed descriptively.

**Results:**

We included five patients (80% female) with a mean age of 56.8 years and mean BMI of 21.8 kg/m^2^. All lesions were confined to the musculofascial layer. Four patients underwent mesh-based reconstruction, while one did not require it. The mean operative time was 144 min, and mean hospital stay was 3.4 days. No intraoperative or postoperative complications were reported. R0 resection was achieved in all cases. No hernia or tumor recurrence was observed during short-term follow-up.

**Conclusion:**

Robotic-assisted resection with or without simultaneous mesh-based reconstruction appears to be a safe and feasible minimally invasive strategy for treating abdominal wall tumors confined to the musculofascial layer. Further prospective studies with larger cohorts are warranted to validate these findings.

## Introduction

The surgical management of abdominal wall tumors presents a dual challenge: achieving an oncologically complete resection (R0) while simultaneously restoring the structural integrity and function of the abdominal wall. Tumors originating from or infiltrating the musculofascial layers require wide excision margins to minimize recurrence risk, which often results in substantial soft tissue defects (1).

Historically, such procedures have been performed via open approaches to allow for adequate exposure, complex dissection, and reconstruction. Open surgery, however, is associated with increased postoperative pain, wound complications, and prolonged recovery time (2). In recent years, robotic-assisted surgery has emerged as a promising alternative for complex abdominal wall reconstructions. The robotic platform offers enhanced visualization, improved instrument articulation, and superior ergonomics, potentially allowing for precise dissection and tension-free mesh placement, even in anatomically challenging locations (3, 4). Early experiences have shown that robotic-assisted abdominal wall surgery may offer advantages in terms of reduced surgical site infections, faster recovery, and shorter hospital stay when compared to open surgery (5).

The aim of this study was to explore the feasibility, safety, and short-term outcomes of this minimally invasive strategy and to contribute to the growing body of evidence on the role of robotic surgery in abdominal wall tumor management.

## Methods

At the Bellinzona e Valli Regional Hospital, a tertiary referral center in Switzerland specialized in robotic abdominal wall surgery, we retrospectively identified from a prospectively maintained database patients who underwent robotic-assisted resection of abdominal wall tumors between January 2020 and April 2025. We included resections of both benign and malignant lesions performed using the Intuitive da Vinci Xi robotic system. Patients were selected for a robotic approach only when the tumor was confined to the musculofascial layer and when both oncologically adequate resection and abdominal wall reconstruction were considered feasible using a minimally invasive technique.

We collected demographic and clinical data, including age, sex, medical history, body mass index (BMI), Charlson comorbidity index, and American Society of Anesthesiologists (ASA) score (6). Additional variables included presenting symptoms, tumor size and location, type of surgical procedure, use of mesh, operative time, length of hospital stay, and postoperative complications, which were classified according to the Clavien–Dindo system (7). All procedures were performed by the same two senior general surgeons, both experts in robotic abdominal wall surgery. After hospital discharge, patients were followed at our outpatient clinic up to 6 weeks postoperatively. Additionally, all patients were contacted by telephone prior to inclusion in the study to assess for any new or ongoing symptoms. Follow-up duration was calculated from the date of surgery to the last clinical assessment.

### Surgical technique

All procedures were performed under general anesthesia with the patient in a supine position. Four robotic trocars were placed laterally or caudally on the abdomen, depending on tumor location, and the robotic system was docked from the right side. Abdominal wall tumors were identified and carefully dissected from surrounding tissues using a monopolar hook and bipolar forceps, with particular attention to preserving adjacent neurovascular structures and achieving an R0 resection. If the tumor was located within or adherent to the musculofascial layer, an *en bloc* resection was performed, including a margin of healthy tissue. Following resection, the resulting defect was assessed, fascial approximation was performed using barbed running sutures, and, if required, the area was reinforced with a synthetic, non-absorbable mesh placed in a sublay or preperitoneal position. Drains were placed selectively.

### Statistical analysis

Data were presented descriptively. Categorical variables were reported as counts and percentages, while continuous variables were expressed as means with standard deviations.

## Results

A total of five patients were identified from our database. Four patients (80%) underwent tumor excision with simultaneous mesh-based reconstruction, while one patient (20%) underwent excision without reconstruction. The mean age was 56.8 ± 17.6 years, and four patients (80%) were female. The mean BMI was 21.8 ± 4.6 kg/m^2^. No intraoperative complications were recorded. The mean operative time was 144 ± 43 min. No postoperative complications occurred. All resections were carried out with free surgical margins (R0). The mean length of hospital stay was 3.4 ± 1.7 days. During the short postoperative follow-up (median Follow-up 4 ± 2 weeks), no case of hernia development or recurrence was recorded. Further clinical details were reported in Table 1. A direct comparison with open surgery was not feasible due to the lack of a comparable cohort at our institution, reflecting the rarity and heterogeneity of these lesions.

**Table 1:** Patient details.

Case	Age	Sex	ASA	BMI Kg/m2	Diagnosis	Tumorsize (mm)	Dimension mesh (cm)	Mesh position (preperitoneal/retromuscular)	En bloc resection	Abdominal wall reconstruction	Operative time (min)
1	78	M	III	23	Metastasis (prostate cancer)	21 × 12 × 12	6 × 6	Preperitoneal polypropylene mesh	Peritoneum, posterior rectus sheath, rectus and transversus musculature	Suture of transversus muscle and rectus sheath	150
2	40	F	I	22.5	Desmoid tumor	30 × 14 × 12	15 × 15	Retromuscolar polypropylene mesh	Peritoneum, posterior rectus sheath, rectus musculature and anterior rectus sheath	Transversus abdominis release, suture of the anterior sheath, suture of the posterior sheath and peritoneum	210
3	65	F	II	18	Metastasis (gastroesophageal junction adenocarcinoma)	44 × 30 × 22	No mesh	None	Peritoneum, transversus musculature, internal oblique musculature and 10th rib	None needed	90
4	37	F	I	28.5	Schwannoma	22 × 13 × 9	8 × 8	Retromuscolar polypropylene mesh	Posterior rectus sheath, rectus musculature and anterior rectus sheath	Suture of the anterior sheath, suture of the posterior sheath and peritoneum	130
5	64	F	III	17	Metastasis (adenocarcinoma of the stomach)	17 × 12 × 11	13 × 13	Preperitoneal polypropylene mesh	Peritoneum, transversus musculature, internal oblique and external oblique muscles	Suture of the external oblique muscle, internal oblique and transversus muscles. Suture of the peritoneum.	140

ASA, American Society of Anesthesiologists scorem; BMI, Body mass index; M, male; F, female.

### Case 1

A 78-year-old man with a history of prostate cancer, previously treated with robotic-assisted radical prostatectomy and pelvic lymphadenectomy followed by radiotherapy, was referred to the surgical outpatient clinic for evaluation of an abdominal wall lesion identified on PET/CT scan. A percutaneous biopsy confirmed the diagnosis of metastatic prostate cancer. The case was discussed at the multidisciplinary tumor board, which recommended surgical resection Figure 1.

**Figure 1:**
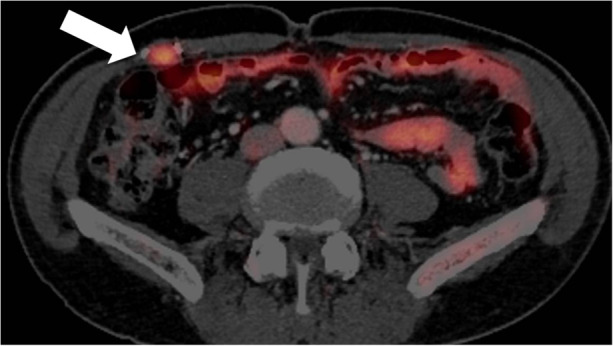
PET/CT scan showing the presence of a metabolic active lesion of the abdominal wall (white arrow).

An *en bloc* excision was performed, including resection of the peritoneum, posterior rectus sheath, rectus abdominis, and transversus abdominis musculature. Reconstruction was achieved by suturing the transversus and rectus sheaths using a V-Loc™ 0 barbed suture. A 6 × 6 cm non-absorbable mesh was placed in the preperitoneal space, and the peritoneum was closed using a V-Loc™ 4–0 suture Figures 2, 3.

**Figure 2:**
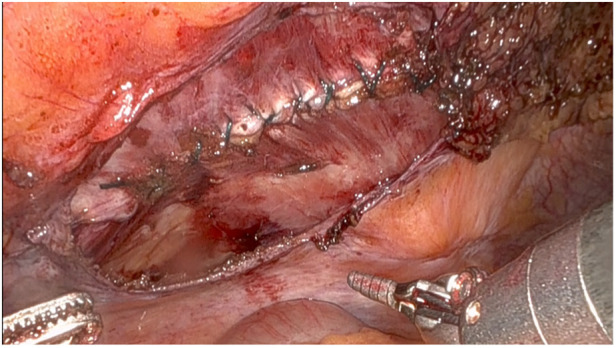
Intraoperative image showing the resection of the tumor and the suture of the fascia.

**Figure 3:**
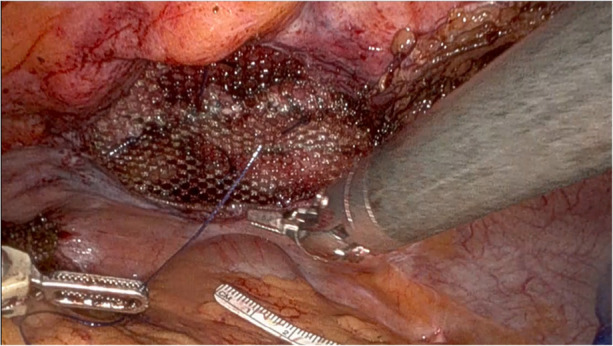
Intraoperative image showing the placement of a non-absorbable mesh on the sutured fascia.

### Case 2

A 40-year-old woman presented to her family physician with pain in the left upper quadrant of the abdomen. On physical examination, a palpable, nodular mass was noted. Ultrasound revealed a homogeneously hypoechoic solid lesion located within the left rectus abdominis muscle. On MRI, the mass appeared hypointense on T1-weighted images and hyperintense on T2-weighted sequences. A percutaneous biopsy confirmed the diagnosis of a desmoid tumor. The case was discussed at the multidisciplinary sarcoma board, which recommended surgical resection.

An *en bloc* excision was performed, including resection of the peritoneum, posterior rectus sheath, rectus abdominis musculature, and anterior rectus sheath. After transversus abdominis release, reconstruction was achieved by suturing the anterior rectus sheath using a V-Loc™ 0 barbed suture, placing a 15 × 15 cm non-absorbable mesh in the retromuscular space, and closing the posterior sheath and peritoneum with a V-Loc™ 3–0 suture.

### Case 3

A 65-year-old woman with a history of gastroesophageal junction adenocarcinoma (Siewert type II) underwent a minimally invasive Ivor-Lewis esophagectomy following neoadjuvant therapy. A subsequent PET-CT scan and biopsy revealed a solitary recurrence in the right abdominal wall. After three months of chemotherapy, follow-up PET-CT confirmed the persistence of the abdominal wall lesion with no evidence of additional metastatic disease. The case was discussed at the multidisciplinary tumor board, which recommended surgical resection.

The mass infiltrated the peritoneum, transversus abdominis and internal oblique muscles, as well as the 10th rib, all of which were resected *en bloc*. The external oblique muscle was spared, as it was not involved by the tumor. Due to the anatomical location of the lesion and the preserved integrity of the external oblique muscle, abdominal wall reconstruction was not deemed necessary.

### Case 4

A 37-year-old woman presented to her family physician with a painful, palpable mass on the right abdominal wall. On physical examination, the mass was tender and non-reducible. Ultrasound revealed a homogeneously hypoechoic lesion within the right rectus abdominis muscle. MRI showed the mass to be isointense on T1-weighted images with strong gadolinium enhancement and multiple ring-like internal structures. Given the high suspicion of a schwannoma, surgical excision was indicated.

The peritoneum was incised well below the lesion. Intraoperatively, the tumor was found to infiltrate the posterior rectus sheath, rectus abdominis muscle, and anterior rectus sheath, but not the peritoneum. En bloc resection was performed. Reconstruction included closure of the anterior rectus sheath with a V-Loc™ 0 barbed suture, placement of an 8 × 8 cm non-absorbable mesh in the retro muscular space, and closure of both the posterior sheath (V-Loc™ 3–0) and the peritoneum (V-Loc™ 4–0).

### Case 5

A 74-year-old woman had previously undergone total gastrectomy for gastric cancer. Due to multiple comorbidities, no adjuvant chemotherapy was administered. A follow-up PET-CT scan and biopsy revealed a solitary recurrence in the right abdominal wall. The case was discussed at the multidisciplinary tumor board, which recommended surgical resection.

The mass was found to infiltrate the peritoneum, transversus abdominis, internal oblique, and external oblique muscles, all of which were resected *en bloc*. Reconstruction was achieved by suturing the external oblique muscle with a V-Loc™ 0 barbed suture, and the internal oblique and transversus muscles with a V-Loc™ 4–0 suture. A 13 × 13 cm non-absorbable mesh was placed in the preperitoneal space, and the peritoneum was closed using a V-Loc™ 4–0 suture.

## Discussion

Robotic-assisted resection, when necessary combined with mesh-based reconstruction, appears to be a feasible and effective surgical approach for treating abdominal wall tumors limited to the musculofascial layer. According to our experience, this technique allows precise dissection and repair while maintaining the benefits of minimally invasive surgery.

Abdominal wall tumors are uncommon entities with a wide range of biological behaviors, from benign to borderline and malignant lesions. The majority are primary soft tissue tumors, while a smaller proportion represent metastatic disease (8, 9). Primary lesions may be identified as palpable masses or incidentally discovered during imaging or clinical examination. Metastatic involvement, particularly from gastrointestinal malignancies, may also present as isolated abdominal wall lesions (9).

Abdominal wall tumors include a wide spectrum of histological types, such as adipocytic, fibroblastic, vascular, and undifferentiated neoplasms. In addition to primary tumors, secondary involvement of the abdominal wall may occur through metastatic spread, most frequently from gastrointestinal cancers, or as a consequence of port-site seeding after minimally invasive procedures (10, 11). It is essential to differentiate neoplastic masses from non-neoplastic entities such as hernias, hematomas, endometriosis, abscesses, seromas, and granulomas. In many cases, a thorough medical history and clinical examination are sufficient. However, imaging modalities such as ultrasound, CT, and MRI, but also preoperative biopsies play a crucial role in confirming the diagnosis and planning surgical intervention when neoplastic processes are suspected (10).

The surgical treatment of abdominal wall tumors entails a dual objective: oncologically sound resection with negative margins (R0) and the restoration of abdominal wall integrity and function (11). Achieving wide tumor-free margins reduces the risk of local recurrence and is especially critical for desmoid tumors and soft tissue sarcomas (1).

Given the extent of resection often required, particularly when involving the musculofascial layer, reconstruction is a key component of treatment. Soft tissue defects may be managed with skin grafts, flaps, or delayed closure techniques. However, when the muscular layer is involved, restoring functional continuity is necessary to prevent herniation, postoperative pain, and impaired mobility (12). Multiple reconstructive techniques have been described, including component separation, autologous flaps, and mesh-based reinforcement. Zhao et al. recently demonstrated the feasibility of immediate mesh-based repair following tumor resection (8). In hernia surgery literature, the use of mesh is associated with a lower risk of recurrence and, when performed minimally invasively, a reduced incidence of surgical site infections (13). The use of mesh in the setting of oncologic abdominal wall resection raises specific considerations, particularly in patients who may require adjuvant therapies. Current evidence does not identify the presence of mesh as a contraindication to radiotherapy; however, it may influence postoperative imaging. In particular, mesh can be visible on CT and MRI and may alter PET signal interpretation (14), which should be carefully considered during radiotherapy planning to ensure accurate target delineation. Additionally, patients undergoing systemic chemotherapy may present an increased risk of infection due to treatment-related immunosuppression (15). Despite these theoretical concerns, no mesh-related infectious complications were observed in our series.

Traditionally, the surgical treatment of abdominal wall tumors has been approached via open surgery. However, in recent years, the adoption of robotic surgery for abdominal wall procedures has expanded considerably, particularly in hernia repair. The robotic platform offers several technical advantages: high-definition 3D visualization, wristed instruments and enhanced precision; factors that support meticulous dissection, hemostasis, and mesh positioning (16).

To our knowledge, this is the first case series reporting robotic-assisted resection of abdominal wall tumors. In all five patients included, negative margins were achieved, and no intra- or postoperative complications were observed. Four patients underwent mesh-reinforced repair; in one case, reconstruction was not required due to the integrity of the external oblique muscle. We believe that the robotic platform's advanced visualization and instrument articulation significantly enhance the ability to perform accurate dissection in confined anatomical planes. Moreover, it allows for effective and tension-free reconstruction with sufficient mesh overlap beyond the defect, which is crucial to prevent incisional hernias (17).

This technique is indicated for tumors confined to the musculofascial layer. Lesions involving more superficial structures, such as the skin or subcutaneous tissue, as well as those extending to deeper visceral components, may require alternative or open surgical approaches. In our series, patients were selected for a robotic approach only when the tumor was limited to the musculofascial layer and when both oncologically adequate resection and effective reconstruction were considered achievable using a minimally invasive technique. Additionally, all resections in our series were performed in a high-volume center by surgeons experienced in robotic abdominal wall surgery. Given the rarity and complexity of these tumors, such procedures should not be attempted at the early stages of the learning curve.

The extremely small sample size and the inherent rarity of these lesions represent a major limitation, significantly restricting the generalizability of our findings. In addition, the retrospective design, the absence of a control group and the short follow-up further limit the strength of any conclusions that can be drawn. Therefore, our results should be interpreted with caution, as they primarily reflect the experience of a highly selected patient cohort treated in a specialized center. While this study suggests that a robotic approach is technically feasible in such cases, larger multicenter studies with adequately powered cohorts are essential to better define its role, validate safety, and assess long-term oncological outcomes.

## Conclusion

In our experience, robotic-assisted resection with simultaneous mesh-based reconstruction is a feasible surgical option to treat abdominal wall tumours confined to the musculofascial layer. Further evidence is needed to assess its safety and advantages.

## Data Availability

The data that support the findings of this study are available on request from the corresponding author.

## Supplementary material

Supplementary Video 1 Presentation of a case of abdominal wall resection and reconstruction with a robotic approach.
